# Visual perceptual learning modulates microsaccade rate and directionality

**DOI:** 10.1038/s41598-023-42768-w

**Published:** 2023-10-02

**Authors:** Shao-Chin Hung, Antoine Barbot, Marisa Carrasco

**Affiliations:** 1 Department of Psychology, New York University, New York, USA; 2 Center for Neural Science, New York University, New York, USA

**Keywords:** Perception, Human behaviour

## Abstract

Microsaccades, incessant “fixational eye movements” (< 1°), are an important window into cognitive functions. Yet, its role in visual perceptual learning (VPL)–improvements in visual discrimination due to practice–remains practically unexplored. Here we investigated whether and how microsaccades change in VPL. Human observers performed a Landolt acuity task for 5 consecutive days and were assigned to the Neutral or Attention group. On each trial, two peripheral Landolt squares were presented briefly along a diagonal. Observers reported the gap side of the target stimulus. Training improved acuity and modified the microsaccade rate; with training, the rate decreased during the fixation period but increased during the response cue. Furthermore, microsaccade direction during the response cue was biased toward the target location, and training enhanced and sped up this bias. Finally, the microsaccade rate during a task-free fixation period correlated with observers’ initial acuity threshold, indicating that the fewer the microsaccades during fixation the better the individual visual acuity. All these results, which were similar for both the Neutral and Attention groups and at both trained and untrained locations, suggest that microsaccades could serve as a physiological marker reflecting functional dynamics in human perceptual learning.

## Introduction

Perceptual learning is the process by which humans improve sensory discrimination due to repetitive practice^1^. Considered a manifestation of neural plasticity, visual perceptual learning (VPL) enables the adult visual system, which is developmentally mature and relatively stable, to yield substantial improvements after extensive training. From a scientific perspective, VPL provides important clues regarding how the mature neural circuitry continues to be gradually refined to efficiently process visual stimuli through training. From a translational perspective, knowledge of VPL provides insights into optimizing learning in real-world applications. For example, VPL has been employed to improve visual performance in people with amblyopia^2–4^, myopia^5^, optical defects^6^, aging^7^, as well as those seeking visual expertise^8,9^ or rehabilitation^10–13^.

VPL arises from neural plasticity in primary visual cortices^14–16^ and a broad network of brain systems, including those related to read-out, attention, feedback, decision, and oculomotor systems^17,18^. Notably, in a recent study investigating feature-based attention and VPL ^19^, we showed that VPL and the oculomotor system are tightly coupled. During VPL, microsaccades were more suppressed for incorrect than correct trials after observers’ responses, and were biased toward the target location prior to its onset. These findings indicate that fixational eye movements may play a functional role in gathering precise feedback information and in anticipating target timing and location.

Fixational eye movements refer to the incessant eye movements across the foveola (~ 1° highest-acuity region at the center of gaze) during fixation. Over the past two decades, microsaccades have been proposed to be physiological correlates of dynamic cognitive processing, including spatial attention^20–22^, temporal attention^23,24^, temporal expectation in the visual, auditory and tactile domains^25–28^, working memory^29,30^ and perceptual learning^19^. Given that VPL has generally been assessed at peripheral locations while observers maintain fixation^15,31,32^, investigating whether and how microsaccades change in VPL tasks can reveal functional dynamics of the oculomotor system during VPL.

Here we used an acuity task to investigate whether and how training with and without exogenous spatial attention modulates microsaccade characteristics –rate and directionality. Prior work established that training in a Landolt acuity task improves acuity performance at trained and untrained locations for both exogenous spatial (Attention group) and distributed (Neutral group) attention conditions^31^. In the current study, we confirmed that perceptual learning benefits generalize to untrained locations in both groups. We additionally found that training decreased the microsaccade rate during fixation and increased it during the response cue period. These microsaccade-rate changes emerged for both the Neutral and Attention groups at the trained and untrained locations. In parallel to the behavioral findings, there was no effect of exogenous attention on microsaccade characteristics and microsaccadic changes generalized across trained and untrained locations. Furthermore, stimulus locations, and in particular the target location, drove microsaccade directionality during the response cue period (i.e., while observers were told which of the two stimulus locations was the target), and this directional bias increased and emerged earlier after training. The present results in a Landolt acuity task, together with recent findings in an orientation discrimination task^19^, provide a tight link between visual and oculomotor processes and suggest microsaccades as a reliable oculomotor correlate in VPL.

## Materials and methods

Note that the Materials and Methods have been reported in detail in two previous studies from our lab; the behavioral task and findings of VPL in Donovan, Shen, Tortarolo, Barbot and Carrasco (2020)^31^ and the microsaccade analysis in Hung & Carrasco (2022)^19^. Here we use the dataset reported in Donovan et al. (2020)^31^ to analyze microsaccades using a similar approach as in Hung & Carrasco (2022)^19^.

### Observers

Twenty-six (19 females; *M* = 24.9 years old; range, 18–35) naïve human observers who had normal or corrected-to-normal vision and had no previous experience with the Landolt square gap discrimination participated in the experiment. Participants were allocated to either the Neutral group (n = 13) or the Attention group (n = 13) before starting the experiment. The experimental protocols were approved by the University Committee on Activities Involving Human Subjects of New York University, and all research was performed in accordance with relevant guidelines and regulations. Informed consent was obtained from all observers.

### Apparatus

The stimuli were presented using Psychophysics Toolbox^33,34^ for MATLAB (The Mathworks, Natick, MA, USA) on a 21-in. gamma-corrected CRT monitor with a resolution of 1280 × 960 pixels and a refresh rate of 85 Hz. An infrared eye tracker system Eyelink 1000 (SR research, Kanata, Ontario, Canada) was used to ensure eye fixation at the center of the display throughout each trial in the experimental sessions. Observers viewed the screen from 114 cm away, using a chin rest to stabilize the head position.

### Stimuli

**Figure 1:**
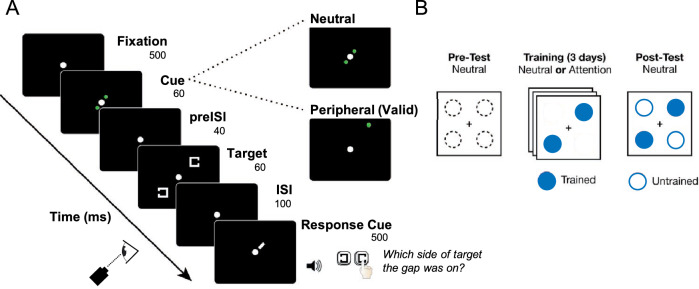
Illustration of the Landolt-C acuity task and design of the visual perceptual learning study. (**A**) Each trial began with a fixation period of 500 ms followed by a 60 ms cue (neutral or peripheral, 100% valid). After a 40 ms preISI, two Landolt squares appeared for 60 ms along one diagonal (top left and bottom right, or top right and bottom left), corresponding to the locations indicated by the neutral cue. Following a 100 ms ISI, a short, white-line response cue appeared for 500 ms at fixation, indicating which of the two stimulus locations was the target. The response cue always indicated the same location as the cue if the cue was peripheral or one of the two diagonal locations if the cue was neutral. Observers were required to indicate, using key presses, whether the gap occurred on the left or right side of the Landolt square at the location indicated by the response cue. Auditory feedback was provided each trial informing observers of the accuracy of their response. (**B**) Schematic illustration of the 5-day VPL experiment. Observers were tested before (Pre-test) and after (Post-test) training at all locations. To isolate the effects of training with spatial attention on VPL, all observers were presented with a neutral cue during both Pre-test and Post-test. For the middle three training sessions, observers were trained with a neutral cue or an attention cue depending on their assigned group. The trained diagonal was counterbalanced across observers (i.e., only top left or bottom right for one observer, only top right or bottom left for another). Thus, each observer had two trained locations, and the two locations in the other diagonal were untrained ^31^.

Stimuli were presented on a black background. In each trial, two Landolt squares (outline squares 1° × 1°) appeared along one diagonal (top left and bottom right, or top right and bottom left), 7.5° from the center fixation dot (radius 0.75°) (Fig. 1A). The cue was either neutral and central (two green dots, subtending 0.1°, 0.7° from fixation, and in intercardinal positions along one diagonal) or valid and peripheral (green circle subtending 0.2° presented 0.95° above the upcoming target). The Landolt squares had seven gap sizes, equally likely, chosen randomly from trial to trial (method of constant stimuli), and ranging from 0.0625° to 0.5°. The two Landolt square stimuli always had equivalent gap sizes, and the side of each stimulus that contained the gap was randomly and independently generated for each Landolt square on a given trial. The response cue was a short 0.42° diagonal white line appeared at fixation, indicating which of the two stimulus locations was the target.

### Landolt acuity task

The experiment was performed with a gaze-contingent display, in which observers were required to maintain fixation at the center (within a 1.5° radius fixation window) until the onset of the response cue period. If an eye-movement outside of this window was detected at any point before the response cue period, the trial would end immediately, and a trial with identical parameters (stimuli and target locations, gap location and size) would be added at the end of the block, ensuring successful completion of all trials within the block without an eye movement. Note that we instructed observers to blink after their responses. During the response cue period, the gaze-contingent display was released, and observers were allowed to blink or move their eyes from the center. The blink rates across observers during the response cue did not differ (*p* = 0.11) between the Pre-test (13.21%) and Post-test (10.25%).

Each trial began with a 500-ms fixation period followed by a 60-ms cue. The cue was either two green circles near the center (neutral) or one green dot above the upcoming target locations (valid and peripheral). Following a brief 40-ms pre-interstimulus interval (preISI), two Landolt squares appeared for 60 ms along one diagonal (top left and bottom right, or top right and bottom left), corresponding to the locations indicated by the neutral cue. Following a 100-ms interstimulus interval (ISI), a response cue appeared at fixation to indicate which of the two stimulus locations was the target. The response cue always indicated the same location as the cue if the cue was peripheral (cue 100% valid) or one of the two diagonal locations if the cue was neutral. After a 500-ms response cue, a middle auditory tone indicated to the observer she or he could respond, using key presses, whether the gap occurred on the left or right side of the Landolt square at the location indicated by the response cue. Observers had unlimited time to respond. Auditory feedback was provided after each trial informing observers of the accuracy of their response (high tone for correct, low tone for incorrect), and text feedback was provided at the end of each block informing observers of their percent correct on that block.

### Experimental procedure

Before the first session (the pre-test), all observers completed 30 trials of a practice task in which a white line oriented left or right was presented left or right of fixation. The practice task had the same procedure and timing as the experiment but with different stimuli (oriented lines) presented at different locations from those used in the main experiment. The purpose of the practice was to familiarize observers with the procedure and timing and reduce procedural learning during the VPL experiment.

The experiment consisted of five sessions that took place on 5 consecutive days at the same or a similar time. The pre-test occurred on day 1 and the post-test on day 5, and three training sessions took place on days 2–4. To isolate the effects of training with spatial attention on VPL, all observers were presented with a neutral cue during both their pre-test (before training) and post-test (after training) sessions.

*Pre-test and Post-test.* The pre-test consisted of 10 blocks of 112 trials per block. Within a single block, two Landolt squares appeared along one diagonal (i.e., top left and bottom right in one block, or top right and bottom left in another). On each trial, the target Landolt square indicated by the response cue was randomly chosen as one of the two Landolt squares that had been presented simultaneously. The tested diagonal alternated between blocks. The pre-test and the post-test were identical for all observers.

*Training.* During the middle three training sessions, observers were randomly assigned to the distributed attention (neutral cue) or focal attention (valid cue) group. Half of the observers were in the Neutral group, in which the neutral cue appeared on all trials. The other half were in the Attention group, in which a valid peripheral cue appeared above the target location on all trials. All observers were trained with stimuli appearing along the same diagonal for all blocks, and the response cue randomly indicated either location as the target. The trained diagonal was counterbalanced across observers (i.e., only top left or bottom right for one observer, only top right or bottom left for another). Thus, each observer had two trained locations, and the two locations in the other diagonal were untrained.

### Data analysis

**Figure 2:**
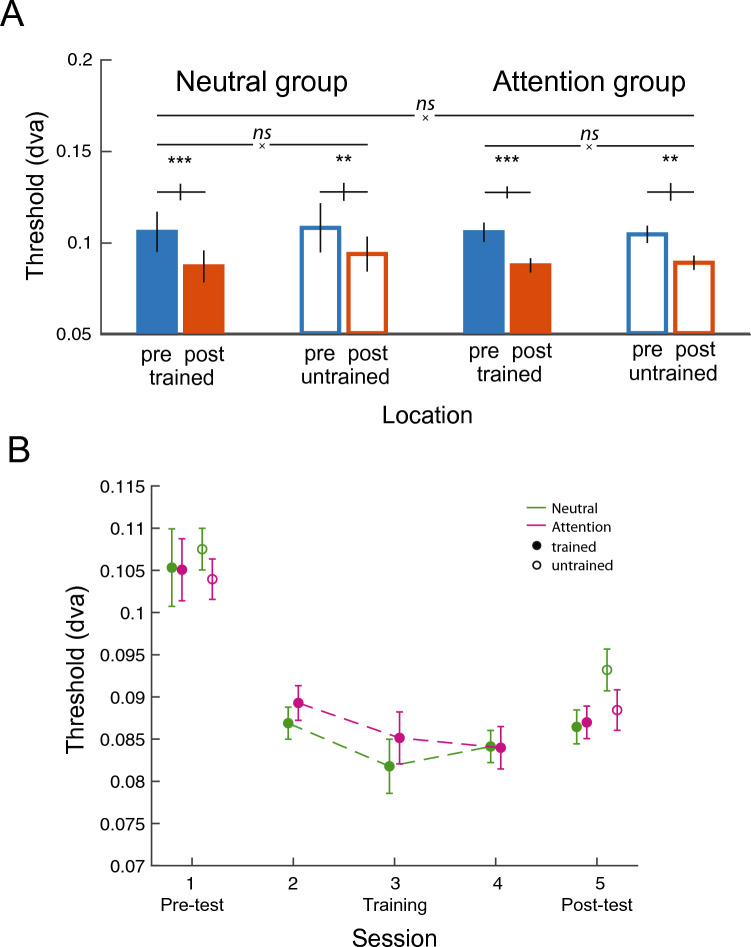
Changes in acuity threshold between Pre-test and Post-test for the Neutral and Attention groups. (**A**) Performance improved at trained locations (solid bars), and learning transfer was present at the untrained locations for both groups (hollow bars). There were no significant differences in learning between the Neutral group (n = 13) and the Attention group (n = 13). (**B**) Changes in acuity threshold across sessions. Performance improved at the trained locations (solid circles) with training, and the improvement transferred to the untrained locations (hollow circles). No difference in learning was observed between the Neutral and Attention groups (n=13 each group). * *p* < 0.05; ** *p* < 0.01; *** *p* < 0.001. Error bars represent ± 1 within-subject SEM ^31^.

*Behavioral data analysis.* For each observer and each of the five sessions, we computed accuracy as a function of gap size and fitted the data with Weibull functions to estimate 75%-correct gap-size thresholds for each diagonal (trained vs. untrained). We averaged the thresholds within each condition on each session and assessed how the change in thresholds after training differed between the Neutral and Attention groups. Figures 1 and 2A have been reported in Donovan et al. (2020).

*Microsaccade detection.* Online eye-tracking was employed throughout the experiment. Raw gaze positions recorded by an Eyelink 1000 eye tracker were converted to degrees of visual angle using the data from a nine-point calibration at the beginning of each session. The first 50 ms of the fixation period was discarded in the analyses to avoid an initial artifact when extracting microsaccades. Blinks were identified based on the Eyelink built-in algorithm, and blink intervals—100 ms before the blink onset and 150 ms after the blink offset—were excluded from the saccade analysis. Saccades were detected using a standard velocity-based algorithm^20^, in which the detection thresholds were determined in two-dimensional (2D) velocity space computed separately for horizontal and vertical components. The threshold per trial was set such that a saccade onset was defined as the point in which its velocity exceeded this trial’s median velocity by 6 or more standard deviations, for a minimum duration of 6 ms. An intersaccadic interval (between the saccade offset and the next saccade onset) of 10 ms was imposed to prevent the detection of overshoots, which sometimes follow saccade offsets and may be erroneously detected as a new saccade. As saccades and microsaccades fall along the main sequence (i.e., saccade amplitudes and peak velocities are highly correlated), lying along the ‘microsaccade-saccade continuum’^35,36^, we defined microsaccades as saccades with an amplitude smaller than 1° of visual angle^25,27,37^. The onset, offset, amplitude, peak velocity, and direction of each microsaccade were computed.

**Figure 3:**
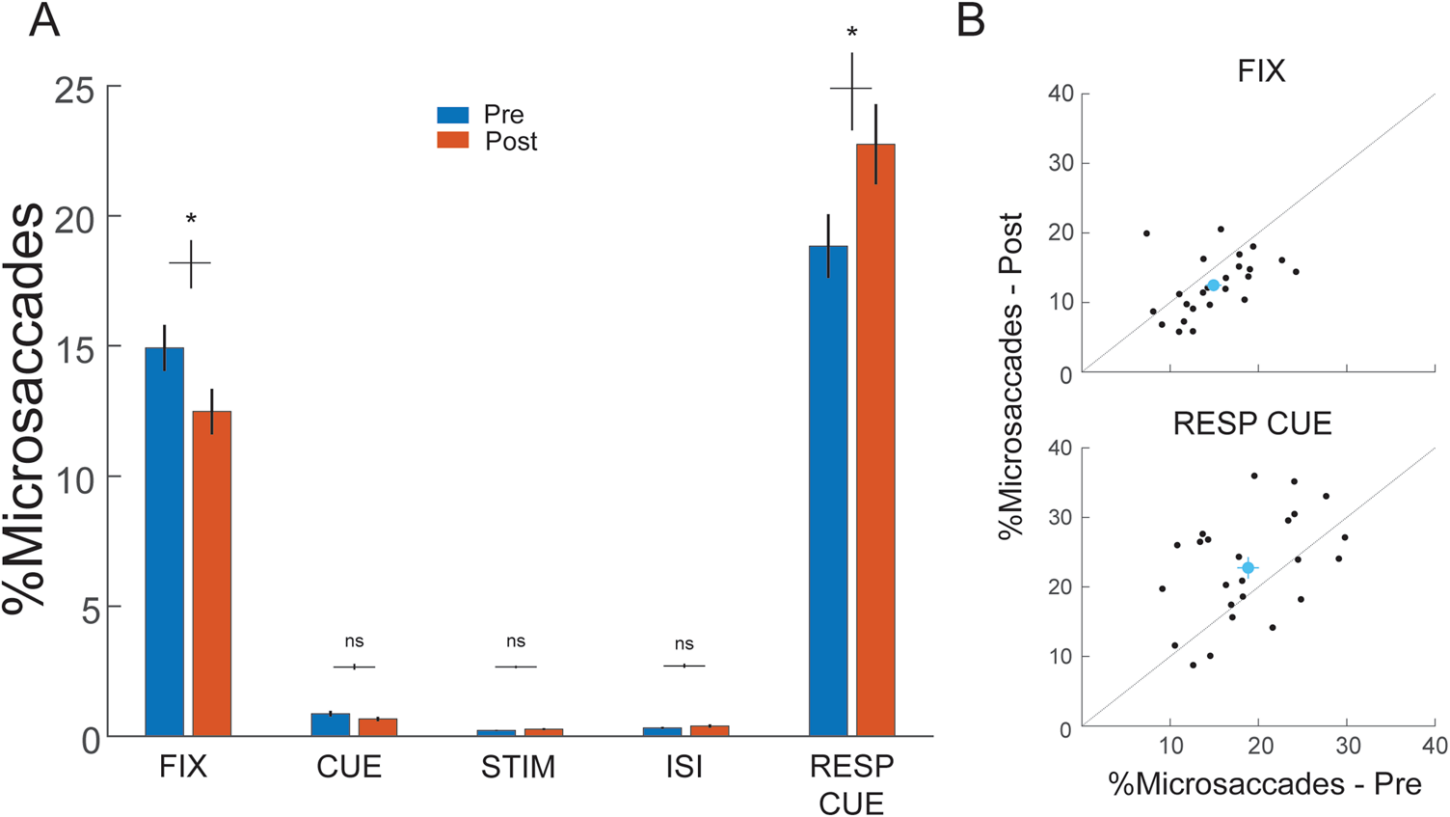
Changes in microsaccade distributions within trials between Pre-test and Post-test. (**A**) The percentage (%) of microsaccades was calculated as the number of microsaccades within a trial segment normalized by each participant’s total number of microsaccades across the trial over Pre-test and Post-test. Data are combined across both Neutral and Attention groups. Microsaccade distribution was comparable in the CUE, STIM and ISI segments between Pre-test (blue bars) and Post-test (orange bars), except for the FIX and RESP CUE segments. After training, the microsaccade percentage was significantly reduced during the fixation period and increased during the response cue period. * *p* < 0.05; ** *p* < 0.01; *** *p* < 0.001. Error bars represent ± 1 within-subject SEM. (**B**) Individual participants’ data at Pre-test versus Post-test in the FIX and RESP CUE segments. Blue dots with lines represent mean and ± 1 SEM.

**Figure 4:**
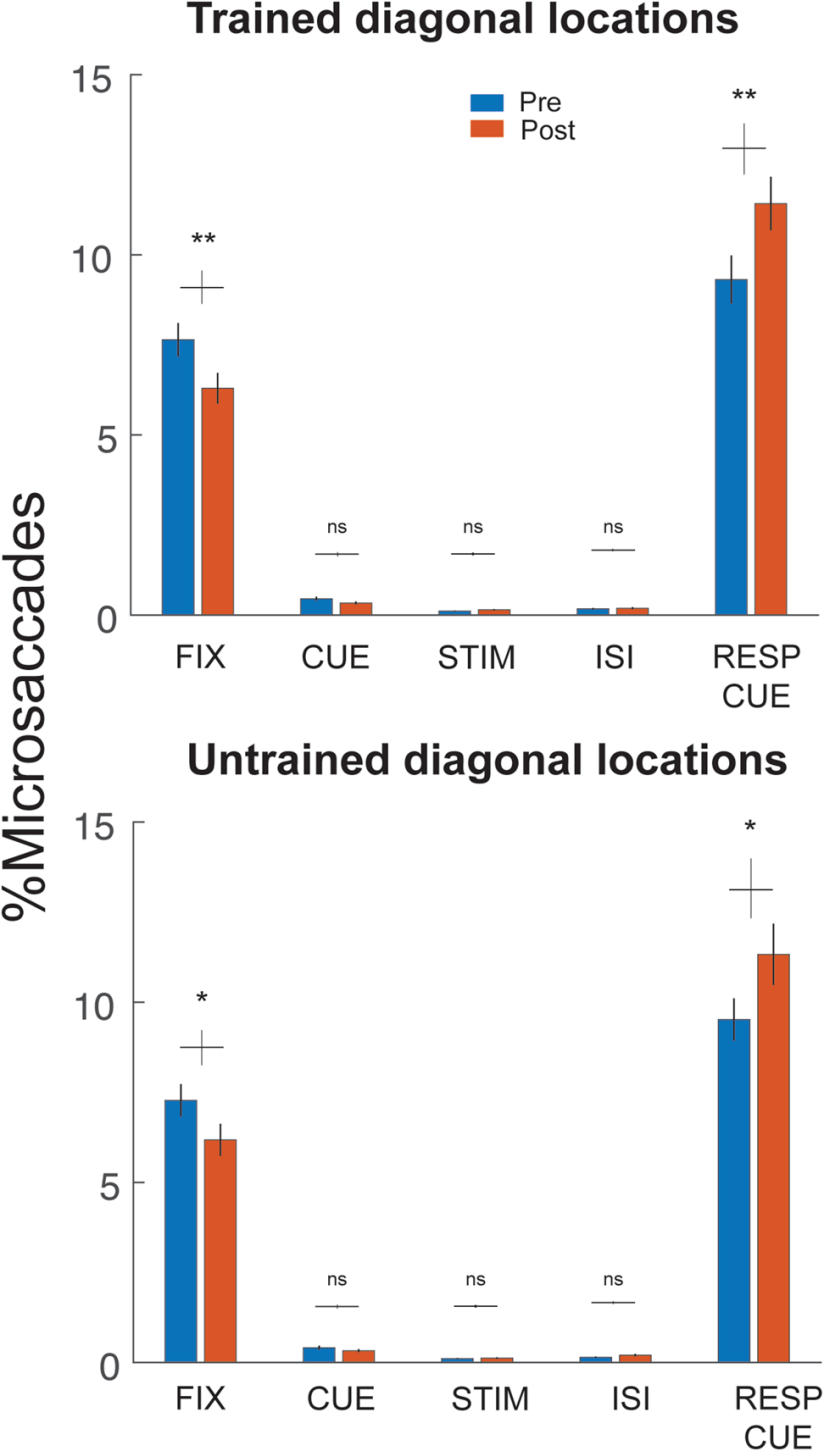
Comparable pattern of microsaccade distributions across the trained and untrained locations. Data are combined across both groups. The microsaccade percentage was reduced during the fixation period and increased during the response cue period in Post-test. This pattern was comparable across trained and untrained locations. * *p* < 0.05; ** *p* < 0.01; *** *p* < 0.001. Error bars represent ± 1 within-subject SEM.

*Microsaccade analysis.* We analyzed the data for 24 observers: all 13 in the Attention group and 11 in the Neutral group (the eye data of the other two observers were lost due to a technical error). To analyze microsaccade distributions across trials, microsaccades were binned based on microsaccade onset within a trial segment (e.g., FIX, CUE, STIM) identified by trial markers. The whole trial sequence was split into five segments—fixation period (FIX, 500 ms), cue and preISI (CUE, 100 ms), stimulus presentation (STIM, 60 ms), interstimulus interval (ISI, 100 ms), and response cue interval (RESP CUE, 500 ms). The percentage of microsaccades for Pre-test and Post-test was computed separately. Percentages were calculated as the number of microsaccades within a trial segment normalized by each observer’s total number of microsaccades across the entire trial by combining both Pre-test and Post-test (Figs. 3 and 4).

**Figure 5:**
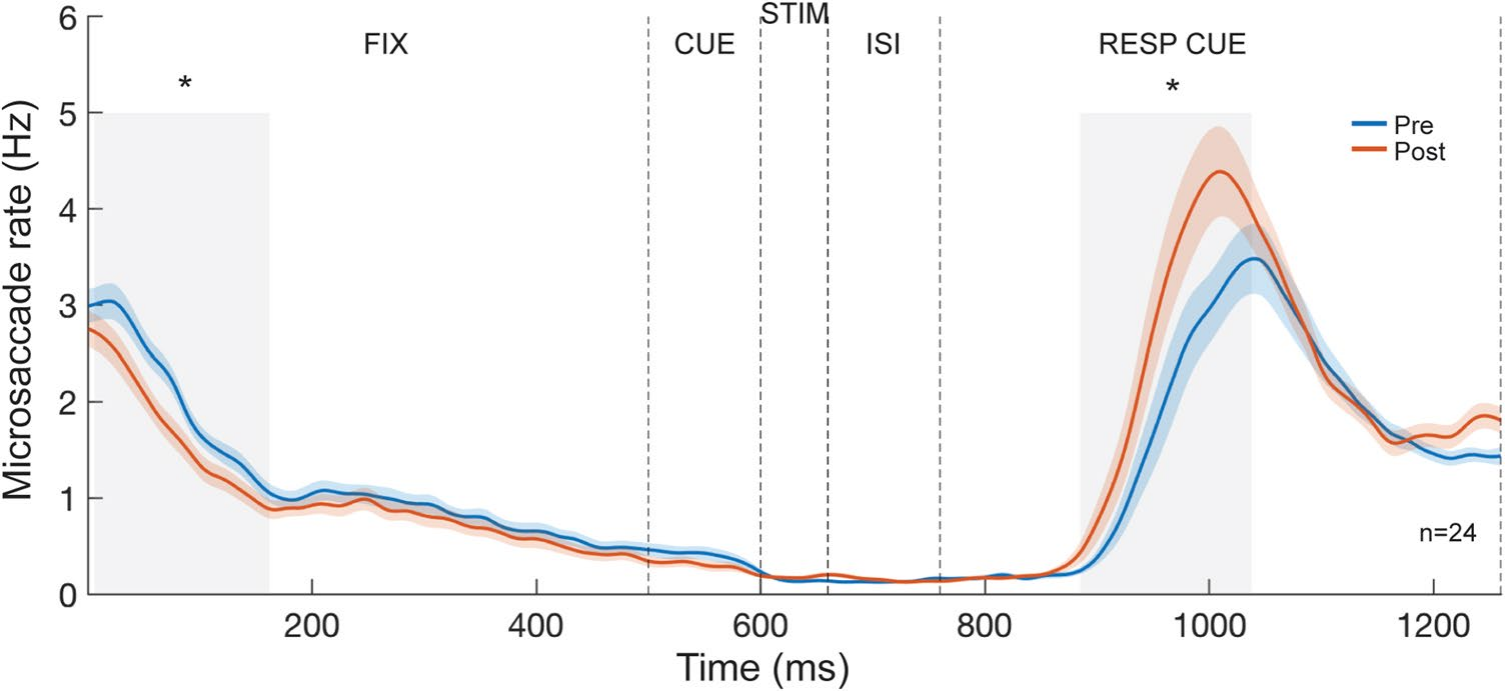
Temporal dynamics of microsaccade rates between Pre-test and Post-test. Data are combined across both groups. Results from a cluster-permutation test revealed that the microsaccade rate decreased during fixation and increased during the response cue after training (gray-shaded areas, both *p* < 0.05). Colored lines and shadings represent mean and ± 1 SEM across observers for each testing session.

**Figure 6:**
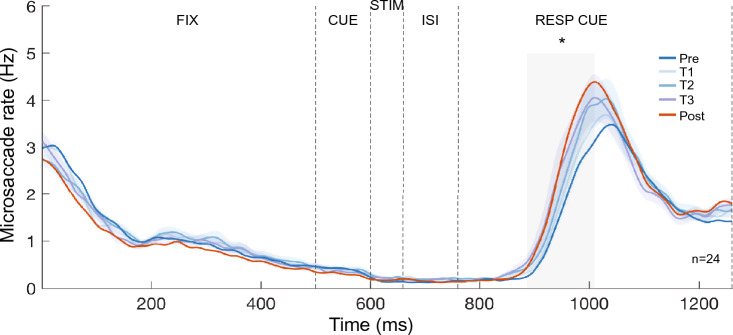
Microsaccade-rate changes emerged during training. Temporal dynamics of the microsaccade rate among the three training sessions (T1–T3). Comparison between T1 and T3 revealed a significant cluster during the response cue (gray-shaded area, *p* < 0.05) period, indicating that the microsaccade-rate changes gradually emerged during training. Colored lines and shadings represent the mean and ± 1 SEM for each training session. Data of the Pre-test and Post-test (without SEM) are also plotted here for comparison.

**Figure 7:**
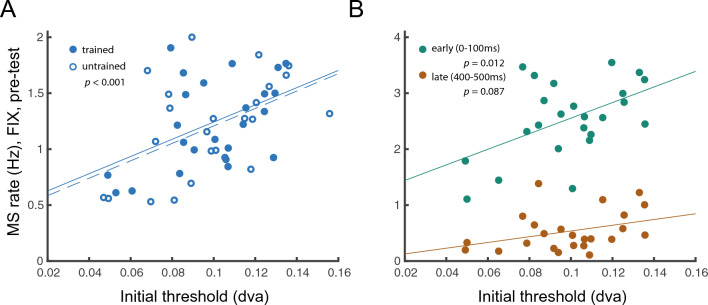
Microsaccade rate during fixation in Pre-test positively correlated with the initial threshold. (**A**) Correlations between the whole fixation period (500 ms) and the initial threshold at the trained and untrained locations (*r* = 0.49*, p* < 0.001). The lower microsaccade rate during fixation, the better initial performance in the Landolt acuity task. (**B**) Correlations between the microsaccade rate during the early (0-100 ms) and the late (400-500 ms) fixation period and the initial threshold. The early period correlated with the initial threshold (*r* = 0.51*, p* = 0.012) but the late period only had a marginal correlation (*r* = 0.36*, p* = 0.087); thus the early period contributed more to the overall correlation (Note y-axes range differs in both figures).

For each observer and session, the microsaccade rate per second for the entire trial sequence was calculated by averaging the number of microsaccades per time point (1 ms) across all trials in each session and multiplying these values by the sampling rate (1000 Hz). (The microsaccade rate was not normalized by subtracting the mean value). The microsaccade-rate time course was then smoothed by applying a sliding Gaussian window of 50 ms (Figs. 5 and 6). We correlated individuals’ microsaccade rate during fixation with their initial threshold (Fig. 7).

**Figure 8:**
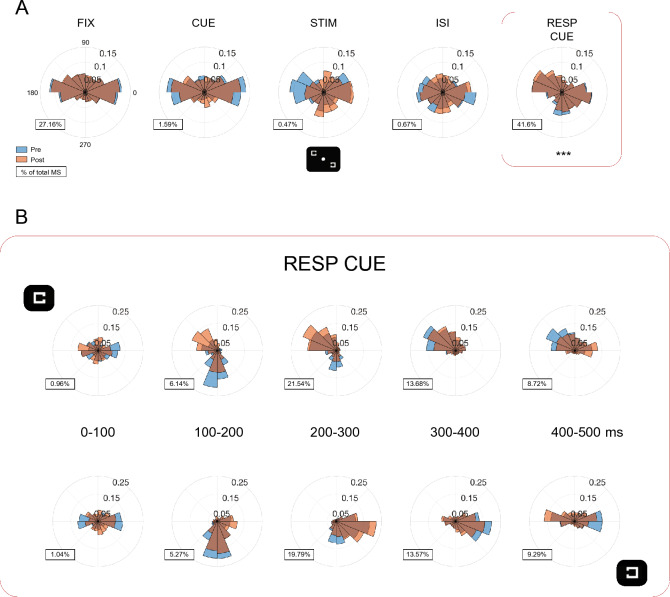
Microsaccade directionality biased toward the target location during the response cue. (**A**) Polar histograms of microsaccade directionality across all trial segments between Pre-test and Post-test. A typical horizontal bias was found during the fixation (FIX) and cue (CUE) periods. Microsaccade directionality was biased toward the stimulus locations (top left and bottom right, illustrated under STIM period) during the response cue, and such bias increased after training (asterisks, *p* < 0.001). Lower left of each polar histogram showed the percentage (%) of microsaccades within a trial segment normalized by each observer’s total number of microsaccades across the trial over the Pre-test and Post-test. (**B**) This directional bias during the response cue was driven by the target location, as microsaccade directionality was prominently skewed to the upper-left (upper row) or bottom-right (lower row) target location. Time course of the directional bias showed that it emerged from 100 ms and became more prominent from 200 ms until 400 ms. Then the microsaccade direction returned to a typical horizontal bias as in the fixation and cue periods. Note the directional bias in Post-test emerged earlier (200–300 ms) than in Pre-test (300–400 ms), indicating a faster readout on target information after training. Lower left of each polar histogram showed the percentage (%) of microsaccades within the response cue period normalized by each observer’s total number of microsaccades across the trial over the Pre-test and Post-test.

To assess microsaccade directionality, which was based on radian angle from the current gaze position, we binned microsaccades in 16 directions on polar histograms (Fig. 8). The proportion of microsaccades in each trial segment was normalized by the total number of microsaccades across the trial over the Pre-test and Post-test. For directionality analysis based on the stimulus location, the direction was normalized by rotating the direction histogram as if the stimulus had been presented on the top left or bottom right visual field.

*Statistics.* For the behavioral results, we assessed the effects of training (within-subject) and attention (between-subject) on Landolt square gap-size thresholds using an ANOVA. Paired t-tests were used to assess the performance changes after training for the trained and untrained conditions within each group.

To assess microsaccade timeseries differences between Pre-test and Post-test across observers in the data, we performed a cluster-based permutation test, which is a nonparametric statistical test that corrects for multiple comparisons at individual time points and determines whether an observed effect is greater than expected by chance^38^. We used a two-sided cluster-based permutation test in which (positive or negative) *t*-values were derived from each time point individually and then obtained clusters comprising contiguous below-threshold (*p* < 0.05) time points. For each permutation iteration, we took the largest cluster mass, which is the largest of the summed absolute *t*-values within a cluster. We shuffled the condition labels for each observer and repeated the same process 1000 times. The largest cluster mass from each permuted dataset thus formed a null distribution of cluster sizes, and we defined a cluster in the data as significant if an observed cluster mass was greater than 95% in the null distribution (*p* < 0.05), while controlling for the false alarm rate for all clusters.

To compare microsaccade directionality between Pre-test and Post-test, we first mapped each microsaccade direction in radians onto a unit circle with a radius of 1. We then calculated the mean unit vector and assessed the difference between the two mean vectors from each dataset (Pre-test vs. Post-test). We performed circular statistics by randomly shuffling angular data in both datasets, regardless of the actual dataset label. This process was repeated 1000 times and the p-value was obtained by the likelihood of the mean difference between the two groups being in the empirical null distribution.

## Results

### Behavior

Observers participated in a 5 day VPL study. Observers in both the Attention and Neutral groups were presented with neutral cues during both the Pre-test and Post-test which assessed their performance at two tested diagonals (Fig. 1B). During the 3 training sessions, the Attention group was trained with a spatial peripheral cue and the Neutral group with a neutral cue near the center (Fig. 1A** right**). Note that the behavior results comparing Pre-test and Post-test sessions (Fig. 2A) have been reported in detail in Donovan et al. (2020, Experiment 1).

We performed a three-way ANOVA with the within-subject factors of training (Pre-test vs. Post-test) and location (trained vs. untrained), and a between-subject factor of group (Neutral vs. Attention) to assess threshold values at Post-test and Pre-test in the Landolt acuity task (Fig. 2A). There was a main effect of training (*F*(1,24) = 38.85, *p* < 0.001), indicating that performance was better at Post-test than at Pre-test. Neither other main effects, nor two-way or three-way interaction were significant (all *p* > 0.1). Paired-sample t-test showed that thresholds at Post-test were significantly lower than at Pre-test for trained and untrained locations, in both the Attention and Neutral groups (all *p* < 0.05).

To assess if there was any difference in learning at trained and untrained locations within the group, we performed two-way ANOVAs between location and training for the Neutral group and the Attention group separately. Neither for the Neutral group (*F*(1,12) = 2.16, *p* > 0.1) nor for the Attention group (*F*(1,12) < 1) the interaction was significant, indicating that learning was comparable between trained and untrained locations for both groups. In Fig.2B, we show the learning curve across the 3 training sessions for the Neutral and Attention groups. As in other VPL studies^32,43^, most of the improvement occurred between the Pre-test and the first training session. Moreover, changes in acuity thresholds during training were highly similar between the Neutral and Attention groups, indicating comparable learning performance. 

### Microsaccade temporal dynamics

The microsaccade-rate time course exhibited a typical pattern across the trial sequence (Fig. 5). The microsaccade rate started from a rate of ~ 3 Hz during the fixation period, and then gradually declined over the CUE period (500–600 ms). The rate was largely suppressed before the stimulus presentation (~ 600 ms) at a rate of ~ 0.2 Hz. This pattern, known as “pretarget inhibition”, indicates observers’ temporal expectation on stimulus presentation in this rhythmic task, consistent with the literature showing oculomotor inhibition prior to a predictable target across sensory modalities^23,25–28^. Because we presented the cue, stimulus, and response cue over a short period (500–760 ms), the microsaccade rate was continuously suppressed over the ISI period (660–760 ms) until the onset of the response cue (~ 760 ms). The long, strong inhibition of microsaccade rate was followed by a prominent rebound to a rate of ~ 4 Hz during the response cue period (~ 900–1100 ms), and then the rate decreased.

### Microsaccadic changes in VPL

We investigated whether the microsaccade distribution and rate changed between Pre-test and Post-test. Because deploying exogenous attention did not significantly affect the microsaccade pattern either during or after training, we combined the data by averaging 24 observers. After training, the microsaccade percentage decreased during the fixation period, whereas it increased during the response cue period (Fig. 3).

We also examined whether microsaccade distributions differ between the trained and untrained locations by separating the corresponding trials. During training, two Landolt squares were presented along one diagonal, and observers were tested on both trained and untrained diagonals before and after training. Training improved acuity both at the trained and untrained diagonals in the Landolt acuity task. We found that microsaccade distributions were comparable across all trial segments between the trained and untrained locations: the microsaccade percentage reduced during the fixation period and increased during the response cue period after training (Fig. 4). Thus, learning-induced microsaccadic changes generalized to untrained locations.

To compare the microsaccade temporal dynamics before and after training, we performed a cluster-based permutation test (see Data analysis: *Statistics*) to assess the difference of microsaccade rates between the Pre-test and Post-test (Fig. 5, blue and orange lines, respectively). The overall microsaccade rates across the trial were comparable (*p* > 0.1) between the Pre-test (~ 1.15 Hz) and Post-test (~ 1.20 Hz). Consistent with the microsaccade distribution analyses (Figs. 3  and 4), after training the microsaccade rate decreased during fixation (6–162 ms, *p* = 0.044) and increased during the response cue (885–1038 ms, *p* = 0.043). Furthermore, the microsaccade-rate increase during the response cue period emerged gradually during the training sessions (886–1010 ms, *p* = 0.042, Fig. 6).

We also analyzed the length of the oculomotor inhibition effect (OMI) by measuring its release time; i.e., the response time (msRT) or latency of the first microsaccade in a time window of 0–160 ms after the stimulus onset. This window did not extend further because the response cue followed immediately after. The mean msRT across observers was 74 ms in the Pre-test and 69 ms in the Post-test (Fig S1) (*p* = 0.073). The marginally faster msRT indicates a shorter oculomotor inhibition (OMI) effect and suggests more efficient visual processing after training.

Further, we explored whether microsaccadic change(s) correspond to behavior change(s) in VPL. We found that neither the reduction of the microsaccade percentage during the fixation period (*r* = 0.27*, p* > 0.1) nor the increase of the microsaccade percentage during the response cue period (*r* = 0.15*, p* > 0.1) correlated with the performance improvement in VPL (Fig S2). However, in the Pre-test, the microsaccade rate during the fixation period was positively correlated with the threshold (*r* = 0.49*, p* < 0.001), in which the lower microsaccade rate during fixation indicated better initial performance in the Landolt acuity task (Fig. 7A). The microsaccade rate during the early fixation period (0–100 ms) correlated with the Pre-test threshold (*r* = 0.51*, p* = 0.012) but the late period (400–500 ms) only correlated marginally (*r* = 0.36*, p* = 0.087); thus, the early period contributed more to the overall correlation (Fig. 7B). The Post-test thresholds were not correlated with the microsaccade rates in the Post-test fixation interval (*r* = -0.20*, p* > 0.1).

### Microsaccade directionality

In addition to microsaccade rate and distribution, we examined microsaccade directionality before and after training across the trial sequence (Fig. 8A). Because of a similar pattern between the trained and untrained diagonals for both groups, we combined and presented data by stimulus locations along the top-left/bottom-right diagonal (black inset under the stimulus period). The polar histograms showed a typical horizontal bias during the fixation, cue and response cue periods. During the stimulus and ISI periods, microsaccade directionality was more uniformly distributed across the polar histograms. Importantly, microsaccade directionality was biased toward the stimulus locations during the response cue, which indicated the target location between the two stimulus locations (Fig. 8A, framed by a red rectangle). This directional bias increased after training, as indicated by the stronger microsaccade directionality in the response cue period in the Post-test than the Pre-test (*p* < 0.001; Fig. 8A, framed by a red rectangle).

Furthermore, we found that this directional bias was driven by the target location, as separating trials by the target location revealed that the directionality was prominently skewed to the upper-left (Fig. 8B, upper row) or bottom-right target location (Fig. 8B, lower row) indicated by the response cue. To explore the dynamics of the directional bias during the response cue (500 ms), we analyzed microsaccade directionality every 100 ms. The time course plot (Fig. 8B) shows that the microsaccade directional bias toward the target locations emerged from 100 ms and became more prominent from 200 ms until 400 ms. Then the microsaccade direction returned to a typical horizontal bias during 400–500 ms, as in the fixation and cue periods. This pattern was consistent between Pre-test and Post-test, but this bias emerged earlier in the Post-test (during 200–300 ms) than in the Pre-test (during 300–400 ms). This difference may reveal a more efficient readout of target information after training. Therefore, microsaccade directionality was more biased toward the stimulus location(s) following training during the response cue period when observers were attempting to retrieve the target information, and training sped up this readout process.

## Discussion

In this study, we reported that VPL modulated microsaccade rate and directionality in an acuity task when observers trained with or without exogenous spatial attention. After training, both groups showed similar improvements at the trained and untrained diagonals. Training decreased the microsaccade rate during the fixation period but increased it during the response cue, and such effects emerged for stimuli presented both at the trained and untrained locations and for both groups. Importantly, training enhanced and sped up the bias in microsaccade directionality driven by the target location during the response cue period. In other words, the microsaccade direction was more biased toward the target location when observers were reading out relevant target information, and this readout process became more efficient following training. Further, microsaccade rates prior to training during simple fixation were predictive of observers’ initial performance in a visual acuity task.

In contrast to the classic location specificity in VPL, performance improvement in the Landolt acuity task transferred to the untrained locations in the Neutral group (Fig. 2). A hallmark in VPL findings is learning specificity to the trained stimulus location, feature and/or eye^39,40^, although mixed findings have been reported^41,42^. In fact, the degree of specificity and transfer depends on numerous factors: task difficulty or precision^43,44^, extent of training^45–47^, adaptation^48^, and covert spatial attention^3,31,49,50^ or feature-based attention^32^. Many VPL studies find partial specificity and partial transfer across tasks. Given that the training curve and improvement were highly comparable between the two groups, we speculate that location transfer in our acuity task could be attributed to two aspects of the task design in which transfer is more likely to occur: (1) two locations were trained instead of just one location^49^ and (2) a constant stimuli procedure, entailing mixed levels of stimuli difficulty instead of only at the threshold level^43^.

Here, the microsaccade distribution differed in the fixation and response cue periods after training. Training reduced the microsaccade percentage in the fixation period and increased it in the response cue period. Although we did not observe a lower microsaccade rate during the stimulus presentation, likely due to a floor effect, the higher microsaccade rate during the response cue period after training may reflect more efficient processing on visual acuity, resulting in less variability of microsaccade latency and thus a more prominent rebound after the stimulus presentation. These microsaccadic changes were ubiquitous across the trained and untrained locations for both the Neutral and Attention groups. Another study finding partial transfer in a visual detection task also reported that silencing of saccade suppression generalized to an untrained location and orientation^51^. Thus, the learning-related saccadic/microsaccadic changes seem independent of task specificity.

The role of microsaccades on spatial covert attention is still being debated^20–22,52–55^. We note that all our results were highly similar for the observers trained with exogenous attention and those trained in a neutral condition. The comparable results between the attention and neutral conditions are in accordance with our previous study deploying feature-based attention^19^, in which our findings were the same for both the participants who trained in the neutral condition and those who trained in the feature-based attention condition. Together, the current results and our previous study^19^ show that deployment of exogenous spatial attention or feature-based attention during VPL does not modulate microsaccadic characteristics.

A general pattern for microsaccades, the oculomotor inhibition effect (OMI), results from a large suppression of microsaccade rates before the stimulus presentation (“pretarget inhibition” for temporally predictable stimuli) followed by a release of inhibition. Compelling evidence has shown that the release time, known as the “response time”, of microsaccades are highly correlated with sensory saliency^56^, language processing^57^, and face familiarity^58^. Overall, the longer the inhibition effect, the longer the processing time. The response time of microsaccades indicated that the release of OMI tended to be faster after training, suggesting that VPL improved visual processing efficiency.

Further, our results highlight the contribution of fixational eye movements on fine spatial vision. We found that prior to training, the lower the microsaccade rate during the fixation period, the lower the initial threshold in the Landolt acuity task. This indicates that the microsaccade rate could serve as a predictor of observers’ initial performance in VPL. This finding is consonant with a recent study showing that other type of fixational eye movements–ocular drifts–in a fixation task can predict visual acuity in healthy adults^59^. The trend of lower microsaccade rate after training is consistent with the idea that this lower rate is related to the improved acuity performance in the present study. These findings suggest that individual spatial acuity is related to characteristics of fixational eye movements (e.g., microsaccades and ocular drifts) in a visual acuity task. This effect seems to be task dependent, as it was not present in our previous study with an orientation discrimination task ^19^.

Critically, the current study reported a prominent directional bias toward the target location during the response cue period, and training enhanced and sped up this bias. Research has shown that visual working memory engages the oculomotor system^29^ as the memory-related neurocircuitry—the hippocampus and associated medial temporal lobe (MTL)—is intricately connected with the generation circuits of saccades and microsaccades^60^. Thus, the directional bias during the response cue could reflect observers’ attempt to retrieve the target information indicated by the response cue before preparing for a keypress, and also supports the evidence that microsaccades can be voluntarily guided based on visual working memory^30^.

Together with the finding of a learning-induced increase of microsaccade rates during the response cue period, we suggest that both microsaccade rates and directionality reveal a unique function of fixational eye movements in retrieving visual representations in perceptual learning. However, given that the response cue was a short line adjacent to fixation pointing to the target, the directional bias could be partially driven by the diagonal visual cue. Future design using an auditory response cue could help disentangle this possibility and reveal to which extent the directional bias was driven by visual working memory.

Our previous study reported a directional bias toward the target location, specifically prior to the target onset, indicating an anticipatory effect of stimulus timing and location on the oculomotor system^19^. In the present study, we found the oculomotor inhibition of microsaccade rates prior to the target, indicating observers’ temporal expectation on stimulus presentation^23,25–28^. However, we did not observe any directional bias prior to target onset as in the previous study^19^. The absence of directional bias in the current study can be explained by its design–we presented the Landolt squares at two locations simultaneously, and thus observers had no expectation on which of the two squares was the target. Instead, we found a clear directional bias when observers were retrieving the target information indicated by the response cue (Fig. 8B). These results indicate that the existence of a directional bias of microsaccades as an anticipatory effect of stimulus location is related to the study design and suggest that both expectation and information retrieval could drive microsaccade directionality toward the target location.

Taken together, the present results on acuity and our recent study on orientation discrimination^19^ provide evidence of tight links between VPL and the oculomotor system, showing that VPL modulates microsaccade distribution, rate and directionality in specific time periods. We found that training reduced microsaccades in the fixation period and increased them in the response cue period. These microsaccadic changes were ubiquitous across the trained and untrained locations irrespective of observers having trained with or without attention. Furthermore, a bias in microsaccade directionality revealed not only temporal expectation but also information readout of the target location. This directional bias indicating information readout during the response cue period became more pronounced and faster after training. In addition, microsaccade rate during simple fixation could predict observers’ initial performance of visual acuity in VPL, suggesting that microsaccades can be an oculomotor marker of fine spatial vision. We suggest that the characteristics of microsaccades could serve as a physiological marker reflecting functional dynamics during information readout in human perceptual learning.

### Supplementary Information

Supplementary Figures.

## Data Availability

The datasets that support the findings of this study are available from the corresponding author upon reasonable request.
