# Supplementary material for: Visual perceptual learning modulates microsaccade rate and directionality
Source: Sci Rep. 2023 Oct 2;13:16525. doi: 10.1038/s41598-023-42768-w (PMC10545683; doi:10.1038/s41598-023-42768-w)
Supplement: Supplementary file 1 — Supplementary Figures. [file 41598_2023_42768_MOESM1_ESM.docx]

**Visual perceptual learning modulates microsaccade rate and directionality**

Shao-Chin Hung, Antoine Barbot & Marisa Carrasco

**Supplementary Online Materials**

**Fig S1. Microsaccadic response times between Pre-test and Post-test.** The response time of microsaccades (msRT), i.e., the latency of the first microsaccade in a time window of 0-160 ms after the stimulus onset. The mean msRT across observers was 74ms in the Pre-test and 69 ms in the Post-test. The marginal difference between Pre-test and Post-test (*p* = 0.073) suggests more efficient visual processing after training, thus a shorter and faster oculomotor inhibition (OMI) effect. Solid circles and bars represent the mean and ±1 SEM for Pre-test and Post-test.

**Fig S2. Correlations between the performance improvement in VPL and the change of microsaccade percentage.** (**A**) Fixation period. The reduction of microsaccade percentage during the fixation period was not correlated with the performance improvement in VPL (*r* = 0.27, *p* > 0.1). (**B**) Response cue period. The reduction of microsaccade percentage during the response cue period was not correlated with the performance improvement in VPL (*r* = 0.15, *p* > 0.1).
